# Filovirus outbreak responses and occupational health effects of chlorine spraying in healthcare workers: a systematic review and meta-analysis of alternative disinfectants and application methods

**DOI:** 10.1101/2024.09.18.24313940

**Published:** 2024-09-19

**Authors:** Luca Fontana, Luca Stabile, Elisa Caracci, Antoine Chaillon, Kamal Ait-Ikhlef, Giorgio Buonanno

**Affiliations:** a: Università degli studi di Cassino e del Lazio Meridionale (UNICAS), Department of Civil and Mechanical Engineering, Cassino, Italy; b: World Health Organization, Geneva, Switzerland; c: University of California, Center for AIDS Research (CFAR), San Diego. USA

**Keywords:** disinfectant, occupational health, outbreak response

## Abstract

**Objective:**

In the context of filovirus outbreaks, chlorine spraying has been the standard for infection prevention and control. Due to potential occupational health risks, public health institutions now recommend wiping, which is labor-intensive and may increase the risk of heat stress for healthcare workers wearing personal protective equipment. This systematic review and meta-analysis quantified the health effects of occupational exposure to chlorine-based products compared to other disinfectants, and the effects of spraying compared to general disinfection tasks (GDTs) like wiping and mopping, in healthcare settings.

**Data sources, design and eligibility criteria:**

MEDLINE, Scopus, and ScienceDirect were searched for studies addressing the association between exposure to disinfectants applied by different application methods and occupational diseases in healthcare settings. Risk of bias was assessed by two independent reviewers using a validated tool.

**Data extraction and synthesis:**

Two reviewers independently screened and performed data extraction and synthesis. A third reviewer resolved disagreements. Meta-analyses were conducted using fixed- and random-effects models based on the Higgins I^2^ statistic.

**Results:**

30 studies investigating chlorine-based products (7,123 participants), glutaraldehyde (6,256 participants), peracetic acid, acetic acid and hydrogen peroxide (4,728 participants), quaternary ammonium compounds (QACs) (9,270 participants), use of spray (4,568 participants) and GDTs (3,480 participants) were included. Most had a cross-sectional design and high risk of bias. Meta-analysis indicates a significant association between respiratory conditions and exposure to chlorine-based products (OR 1.71, 95%CI 1.41-2.08), glutaraldehyde (OR 1.44, 95%CI 1.14-1.81), QACs (OR 1.30, 95%CI 1.06-1.60), use of spray (OR 2.25, 95%CI 1.61-3.14) and GDTs (OR 2.20, 95%CI 1.66-2.90). The relative odds ratio (ROR) of respiratory conditions for chlorine-based products compared to QACs was 0.76 (95%CI 0.62-0.94). The ROR for the use of spray compared to GDTs was 0.98 (95%CI 0.74-1.29). Strengths include evaluating respiratory health risks of disinfectants, applying a validated tool, using both fixed- and random-effects models, and comparing pooled effect sizes. Limitations include high risk of bias for the majority of included articles, varying confounder adjustments, underreported non-respiratory outcomes, and unspecified disinfectants and PPE use for spray and GDTs articles.

**Conclusion:**

Chlorine-based disinfectants significantly increase respiratory risk compared to QACs. Sprays and general disinfection tasks present similar risks. Our findings advocate for using less hazardous products like QACs, rather than banning sprays in filovirus outbreak responses to enhance disinfection safety.

**Prospero registration number:**

CRD42023479363

## 2. INTRODUCTION

The *Filoviridae* family comprises two genera, Ebolavirus and Marburgvirus, both of which have caused numerous outbreaks with high fatality rates over the past few decades^1^. Human-to-human transmission occurs through contact with an infected person’s body fluid. Infection prevention and control guidance from organizations like the U.S. Centers for Disease Control and Prevention and the World Health Organization (WHO)^2,3^ recommended spraying 0.5 % chlorine on both animate and inanimate objects, including healthcare workers (HCWs) wearing personal protective equipment (PPE) who were in direct or indirect contact with the virus.

Over the last few years, the use of chlorine spraying as a disinfection method has gained considerable attention due to its potential occupational health risks^4,5^, leading public health institutions to reconsider their recommendations. WHO now bans the direct spraying of HCWs and recommends chlorine wiping as the preferred method for surface disinfection, sidelining the once-favored spraying^6^. However, the systematic review supporting this decision did not identify any evidence for the differential effects of spraying versus wiping on efficacy or adverse health events in filovirus settings. Consequently, the recommendation is primarily based on expert judgment and, as stated in the recently published guideline, on evidence of very low certainty^6^.

It is important to acknowledge the attributes that initially made chlorine spraying an attractive disinfection method:

- Economic viability: Chlorine remains a low-budget disinfectant solution.
- Efficiency: Wiping is labor-intensive and time-consuming, making spraying a logistically attractive option. This is particularly relevant for HCWs wearing impermeable PPE in hot and humid environments, such as during the outbreaks in West and Central Africa, as it increases the risk of heat stress and heat-stress-related injuries^7^. Moreover, the discomfort and reduced work efficiency caused by wearing PPE in high-temperature environments can lead to potential health issues for staff^8^.

Given these contrasting viewpoints and the absence of evidence supporting the decision to change guidance, coupled with the critical importance of effective and safe disinfection procedures during filovirus outbreaks and the safety of HCWs and patients, we conducted a systematic review. Our aim was to characterize and, when possible, quantify the health effects of occupational exposure to chlorine-based products compared to other disinfectants and application methods, such as spraying and wiping, in healthcare settings, extending beyond filovirus treatment centers.

## 3. METHODS

### 3.1. Search strategy and eligibility criteria

This systematic review was conducted following the preferred reporting items for systematic reviews and meta-analysis (PRISMA-P) protocol^9^. The research protocol was registered a priori with the PROSPERO database (PROSPERO ID: CRD42023479363).

We searched MEDLINE, Scopus, and ScienceDirect, on 15 November 2023, and re-ran the search on 1 March 2024, for full-text articles in English, without restricting the publication period. Additional studies were searched manually by examining the references of the included studies. Unpublished studies were not sought. The search strategy used free-text terms reflecting the eligibility criteria and was adapted for each database with ‘MeSH’ filters where appropriate (search strings are available in supplementary materials, Table S1).

Eligibility criteria were based on the population (P), exposure (E), comparison (C), outcome (O), and study design (S) approach^10^ as follows: P: HCWs; E: occupational exposure to chlorine-based disinfectants applied by spray; C: HCWs exposed to different disinfectants and/or application methods; O: occupational diseases such as respiratory diseases, symptoms, lung dysfunction, or skin and eye symptoms; and S: case reports and series, cohort studies, case-control studies, cross-sectional studies, and experimental studies. Qualitative studies, abstracts, conference papers/posters, reviews, letters, editorials were excluded. To maximize the number of articles, there were no restrictions on the publication date. The full list of inclusion/exclusion criteria is available in the supplementary materials table S2.

### 3.2. Study selection and data extraction

Two authors (LF and EC) independently assessed the retrieved references against the eligibility criteria and performed data extraction. In cases of disagreement, consensus was reached by consulting a third reviewer (GB or LS). Mendeley was used as reference management software. The reasons for exclusion were recorded only during the full-text review. Data extraction and synthesis were conducted using a predesigned sheet (Table S3), which captured detailed information on study characteristics, sample characteristics and recruitment, methods of exposure and outcomes assessment, and findings.

### 3.3. Risk of bias within studies

Two authors (LF and EC) independently assessed the risk of bias as high, low, or unclear against eight domains of bias, using a tool previously used for other occupational health reviews^11–13^. Disagreements were resolved after discussion with a third reviewer (LS). The hybrid tool uses Scottish Intercollegiate Guidelines Network 2004^14^ and Critical Appraisal Skills Program 2004/2006 assessment tools^15^. The tool can be found in the supplementary materials, Table S4.

### 3.4. Summary

The primary outcomes assessed in this review were the associations between occupational exposure to disinfectants applied by various methods and the incidence of occupational diseases.

### 3.5. Meta-analysis

Studies were categorized into groups based on the specific intervention assessed: four groups for disinfectants (chlorine-based products; glutaraldehyde; peracetic acid [PAA], acetic acid [AA] and hydrogen peroxide [HP]; and quaternary ammonium compounds [QACs]), two groups for application methods (use of spray and general disinfection tasks [GDTs], defined as any other disinfection-related activities except spraying such as wiping, mopping, disinfection of patient rooms, furniture surfaces, equipment, and preparation and dilution of products), and one group for mitigation measures such as indoor ventilation and PPE which were included for completeness. Health outcomes were categorized into clusters based on their relevance as respiratory, ocular-nasal, neurological, gastrointestinal, reproductive, and skin conditions. Meta-analysis was performed when at least two primary studies with similar exposures and health outcomes were available. When a study reported multiple outcomes within the same category, they were treated as independent measures to provide a comprehensive assessment. The fixed-effects and random-effects models were employed to generate pooled effect sizes across the studies. The decision to use either the fixed- or random-effects model results was determined based on the Higgins I^2^ statistic. Significant heterogeneity among studies was considered present when I^2^≥50%^16^. Parameters were estimated using the Restricted Maximum Likelihood method with the metafor R package^17^. Heterogeneity was quantified using the I^2^ statistic and the tau-squared (τ^2^) statistic. Egger’s test and funnel plots were used to assess publication biases.

Meta-regression was conducted to examine the impact of study design and sample size on the observed heterogeneity. The analysis assessed residual heterogeneity and tested the significance of the moderators using the QM statistic, while the R^2^ statistic quantified the proportion of heterogeneity explained by the model. To address potential concerns of non-independence, evaluate the robustness, and determine the importance of individual studies on the overall meta-analysis results, a leave-one-out sensitivity analysis was conducted. To compare the risks associated with chlorine-based products to other disinfectants, and spraying to general disinfection tasks, the relative odds ratio (ROR) was calculated. Statistical analyses were performed using R version 4.3.2. More details are provided in the supplementary materials.

## 4. RESULTS

**Figure 1.**
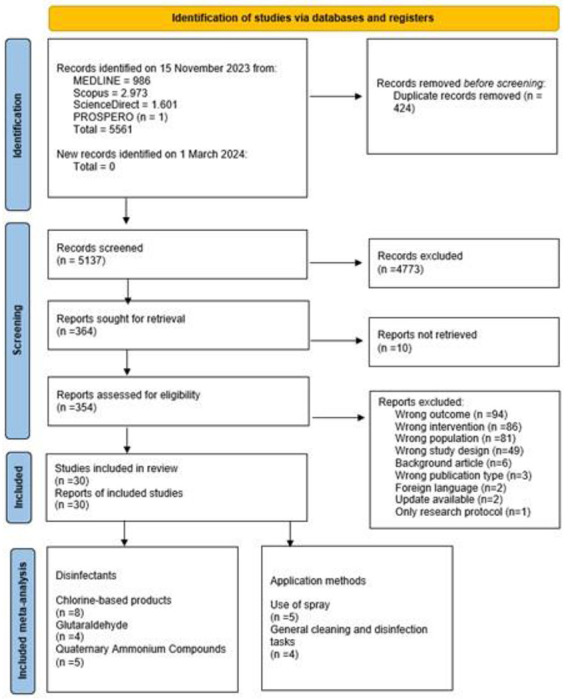
Flow diagram of literature search and selection criteria adapted from Preferred Reporting Items for Systematic Reviews And Meta-Analyses (adapted from Moher et al^18^)

**Figure 2.**
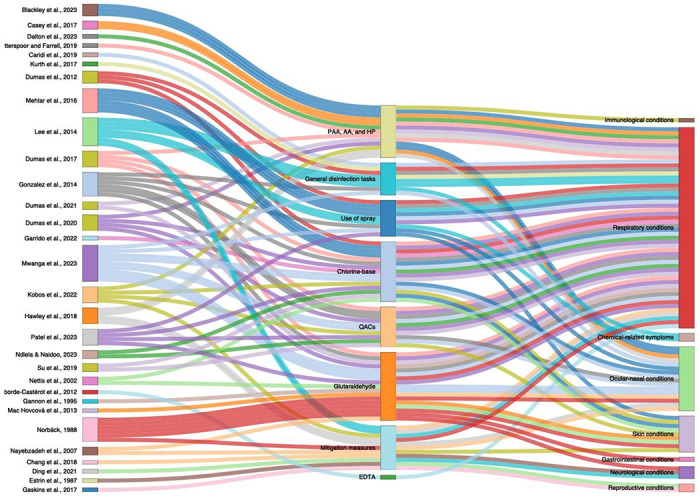
Alluvial plot displaying the clustering of studies based on the intervention or exposure assessed and the associated health outcomes. Each study is linked to specific interventions or exposures, which are then connected to various health outcomes.

From the electronic databases search, 5,561 articles were retrieved. After removing duplicates, 5,137 articles remained for title and abstract screening. Following this, 364 articles were eligible for full-text review. Despite searching, the complete text of 10 publications could not be found, so those were excluded from the review. After applying eligibility criteria, 30 studies were included (Figure 1). Data synthesis and categorization for the included studies are available in Table 1 and Figure 2, respectively. Quantitative data are available in supplementary materials Table S5. Individual reasons for study exclusion are available in Table S6. Among the included studies, 16 were cross-sectional, six were cohort, two were mixed-method experimental and observational, two were case-control, two were case series, and two were case reports. Among the studies, seven had a low risk of bias, and 23 had a high risk of bias (Table S7).

### 4.1. Chlorine-based products

Eleven studies examined occupational health effects of chlorine-based product exposure (Table 1). Dumas et al. (2017) assessed the impact of exposure to various disinfectants on asthma management among nurses. Through a survey, the study found that bleach exposure was associated with suboptimal asthma control (Odd Ratio [OR] 1.55, 95%CI 1.14–2.10, p=0.02)^28^. Dumas et al. (2012) and Dumas et al. (2020), using a job-task-exposure matrix, found no association between bleach and asthma incidence^25,26^. Gonzalez et al. investigated the increased incidence of asthma among HCWs and its potential association to cleaning and disinfection products. Through questionnaire, physical examination and immunoglobulin E (IgE) assays, the authors concluded that chlorinated product/bleach exposure was not significantly associated with reported new-onset asthma (OR 2.08, 95%CI 0.86–5.00, p=0.1)^33^. Kobos et al. characterized the occurrence of skin and allergy symptoms related to the use of cleaning and disinfectant products among HCWs. Using a survey methodology, the authors found that bleach use was associated with skin disorders and allergic reactions (OR 1.79, 95%CI 1.14–2.80, p<0.05)^35^. During the 2014-2016 Ebola outbreak, spraying environments and individuals, including HCWs, with chlorine was common. Mehtar et al. investigated the health outcomes associated with chlorine exposure. They conducted a cross-sectional survey, interviewing 1,550 volunteers, including 500 HCWs, 550 Ebola survivors, and 500 quarantined asymptomatic Ebola contacts. Results indicated that multiple exposures were significantly associated with increased respiratory (OR 32, 95%CI 22–49, p<0.001), eye (OR 30, 95%CI 21–43, p<0.001), and skin conditions (OR 22, 95%CI 15–32, p<0.001)^40^. Mwanga et al. investigated the association between cleaning agents and health conditions among HCWs. A significant association was found between bleach exposure above 100 minutes per week and work-related ocular-nasal symptoms, particularly in those cleaning medical instruments (OR 2.37, 95%CI 1.30–4.34, p<0.001). Conversely, the same exposure to bleach was not significantly associated to work-related asthma (OR 1.16, 95%CI 0.49–2.75, p>0.5)^41^. Ndela et al. investigated occupational exposure to cleaning agents among healthcare cleaners and the risk of respiratory conditions. Through questionnaires and clinical evaluations, they found that exposure to chlorine and bleach was not associated with various respiratory conditions^43^. Su et al. investigated asthma diversity and severity among HCWs related to cleaning and disinfecting activities (CDAs). Using survey data and data reduction techniques, they categorized HCWs by asthma symptoms and CDA exposure. Participants were grouped via hierarchical clustering based on asthma symptom/care variables and product applications. The cluster associated with chlorine product use showed a strong association with “undiagnosed/untreated asthma” (OR 3.11, 95%CI 1.46–6.63, p=0.003) and “asthma attacks/exacerbations” (OR 2.71, 95%CI 1.25–5.86, p=0.011)^48^. Garrido et al. assessed work tasks and cleaning/disinfecting agents associated with respiratory symptoms. After adjusting for age and sex bleach was not significantly associated with tightness in the chest^31^. Patel et al. examined the associations of disinfection tasks and products with work-related asthma symptoms in HCWs. After adjusting for confounding factors, the authors concluded that bleach was associated with new asthma onset (OR 1.91, 95%CI 1.10–3.33, p<0.05)^47^.

### 4.2. Chlorine-based products meta-analysis

**Figure 3.**
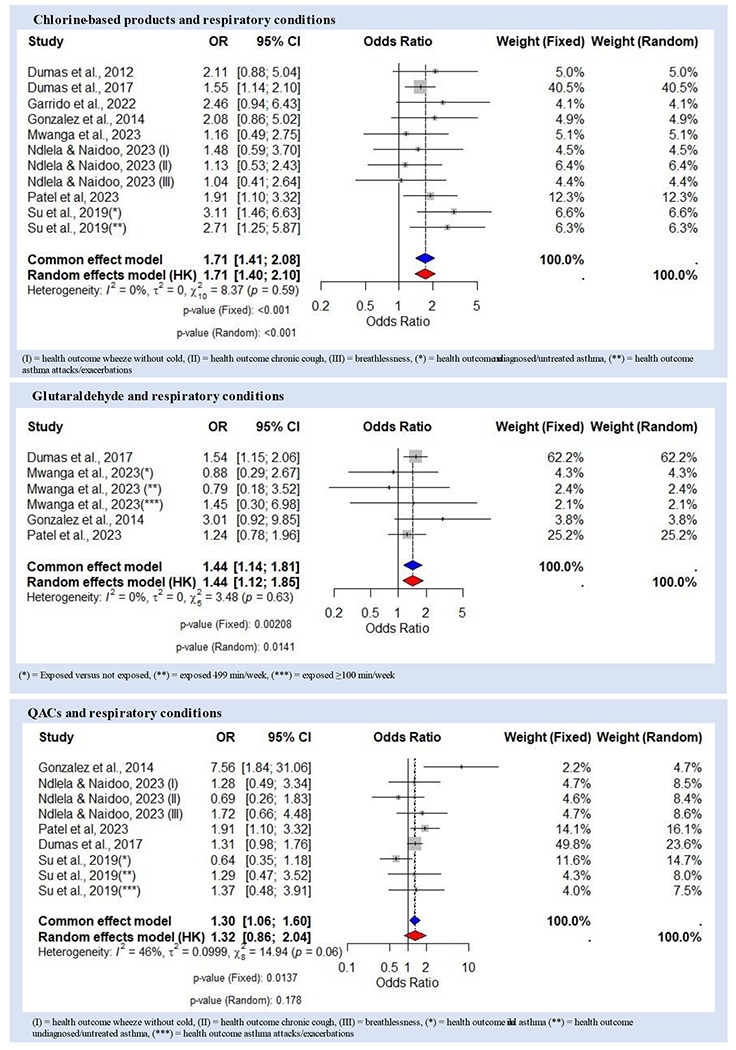
Forest plot showing odds ratios (OR) with 95% confidence intervals (CI) for respiratory conditions associated with different disinfectants. The blue diamond represents the overall common effect estimate, while the red diamond represents the random effect estimate.

The meta-analysis of chlorine-based products’ effects on respiratory conditions included eight studies with 7,123 individuals, addressing 11 adverse effects. Three studies were excluded, with reasons detailed in Table S8 of the supplementary material. The fixed-effect model yielded an OR of 1.71 (95% CI 1.41–2.08, p<0.001). The random-effects model showed an OR of 1.71 (95% CI 1.40–2.10, p<0.001), with non-significant heterogeneity among the included studies (I^2^=0%, τ^2^=0, p=0.59) (Figure 3).

The symmetrical funnel plot and the Egger’s test (p=0.56) suggest no substantial publication bias (Table S9). The meta-regression analysis showed a negative coefficient for cross-sectional study design, suggesting that studies with this design type reported a slightly lower effect estimate (Table S10). The leave-one-out analysis indicated the overall effect estimate remained stable and significant, with the fixed-effect model OR ranging from 1.64 to 1.84 (Table S11).

### 4.3. Glutaraldehyde

The occupational risk associated with glutaraldehyde has been evaluated in nine studies (Table 1).

Gannon et al. examined occupational asthma in HCWs exposed to glutaraldehyde, assessing eight workers from endoscopy units and x-ray darkrooms. They conducted serial measurements of peak expiratory flow (PEF) and specific bronchial provocation tests. Glutaraldehyde levels were monitored with personal and static short- and long-term air samples during challenge tests and in the workplace. Occupational asthma was confirmed in seven workers, all showing PEF records indicative of occupational asthma and positive bronchial challenge tests to glutaraldehyde. The mean glutaraldehyde level during challenge tests was 0.068 mg/m^3^, about one-tenth of the short-term occupational exposure standard of 0.7 mg/m^3^. The authors concluded that glutaraldehyde can cause occupational asthma at levels much lower than current exposure limits^30^. Gonzalez et al. found that glutaraldehyde exposure was not significantly associated with reported new-onset asthma (OR 3.01, 95%CI 0.92–9.86, p=0.061)^33^. Nayebzadeh et al. evaluated how work practices and ventilation systems influenced peak exposure to glutaraldehyde. They collected 42 personal air samples in five hospitals, observing and recording work practices during sampling. The geometric mean concentration of all samples was 0.025 ppm. In areas with poor or unsafe practices, concentrations were higher, with geometric means of 0.05 ppm and 0.08 ppm. All concentrations were below the occupational exposure limit of 0.2 ppm. The study highlighted that work practices and ventilation significantly affect glutaraldehyde exposure levels^42^. Norbäck examined the health impacts of glutaraldehyde exposure among HCWs. Exposure was measured in the breathing zone using sorbent tubes and liquid chromatography. Intermittent exposure levels were below the Swedish occupational exposure limit. In spite of the low exposure, the exposed group exhibited a significantly increased frequency of skin and airway symptoms, as well as headaches, in comparison with the unexposed group^45^. Dumas et al. (2017) assessed the impact of exposure to various disinfectants on asthma management among nurses. The authors concluded that exposure to glutaraldehyde was associated with suboptimal asthma control (OR 1.54, 95%CI 1.15–2.06, p=0.02)^28^. Dumas et al. (2020) and Dumas et al. (2021) found no association between glutaraldehyde and asthma incidence^26,27^. Mwanga et al. investigated the association between cleaning agents and health conditions among HCWs. A significant association was found between glutaraldehyde exposure above 100 minutes per week and work-related ocular-nasal symptoms (OR 3.69, 95%CI 1.30–10.45, p<0.05). Conversely, the same exposure was not significantly associated to work-related asthma (OR 1.45, 95%CI 0.30–6.95, p>0.5)^41^. Similarly, Patel et al. found that glutaraldehyde was not significantly associated with new asthma onset^47^.

### 4.4. Glutaraldehyde meta-analysis

The meta-analysis of glutaraldehyde exposure on respiratory conditions included four studies with 6,256 individuals, addressing six adverse effects. Five studies were excluded, with reasons detailed in Table S8. The fixed-effect model yielded an OR of 1.44 (95% CI 1.14–1.81, p<0.01). The random-effects model showed an OR of 1.44 (95% CI 1.12–1.85, p=0.014), with non-significant heterogeneity among the included studies (I^2^=0%, τ^2^=0, p=0.63) (Figure 3).

The symmetrical funnel plot and the Egger’s test (p=0.69) suggest no substantial publication bias (Table S9). The meta-regression analysis did not identify any significant moderators impacting the overall effect (Table S10). The leave-one-out analysis indicated the overall effect estimate remained stable and significant, with the fixed-effect model OR ranging from 1.28 to 1.51 (Table S11).

### 4.5. Peracetic acid, acetic acid and hydrogen peroxide

Nine studies assessed the occupational risk related to products containing PAA, AA and HP (Table S6). Dalton et al. measured eye and respiratory irritation from a PAA-based disinfectant and HP in a controlled chamber and hospital’s departments. Volunteers wiped surfaces with PPA and HP wetted cloths for 20 minutes. The authors found that, although air sampling indicated 95^th^ percentile breathing zone concentrations of 667 ppb, volunteers showed no significant increases in IgE or inflammation over 75 test days^23^. Casey et al. evaluated health risks from a disinfectant containing PAA, AA, and HP. Among 163 HCWs, 49 air samples were analyzed. All HP and AA levels were below OSHA’s Permissible Exposure Limits (PELs), while no PEL exists for PAA. Workers in department with the highest exposure levels had a higher prevalence of watery eyes (OR 2.88; 95%CI 1.18–7.05, p<0.05) and over three times the rate of current asthma compared to the U.S. population^21^. Dumas et al. (2017), Dumas et al. (2020) and Dumas et al. (2021) found no association between hydrogen peroxide and asthma incidence^26–28^. Otterspoor and Farrell compared three disinfectant solutions in a hospital operating theatre. A staff survey found no respiratory issues related to PAA, indicating a lower risk of respiratory irritation compared to chlorine-based and HP-based disinfectants^46^. Hawley et al. assessed health and exposure in a hospital using a new sporicidal product with HP, PAA, and AA. Among 50 participants, 49 full-shift air samples were collected. Despite low exposure levels, 44% of cleaning staff reported eye symptoms, 58% upper airway symptoms, and 34% lower airway symptoms, with significant correlations to HP, PAA, and the mixture of all three chemicals^34^. Blackley et al. examined health impacts of sporicidal products containing HP, PAA, and AA. In 2018, 56 personal and area air samples were collected from cleaning staff. Significant associations were found between chemical exposures and eye and airway symptoms both cross-shift and over four weeks, despite levels being below US OELs. The study recommended engineering, administrative, and PPE controls to reduce chemical exposure^19^. Kobos et al. estimated that HCWs using cleaning products containing HP were from 2-fold to 6-fold more likely to report allergic reactions compared to the respondents who did not use those products^35^.

Meta-analysis was not performed due to the absence of primary studies with similar exposures and outcomes.

### 4.6. Quaternary ammonium compounds

Eight studies assessed the occupational health risks related to QACs (Table 1).

Gonzalez et al. found a significant risk of asthma among HCWs linked to QACs in cleaning products, with an OR of 7.5 (95%CI 1.84–31.1, p<0.05) for asthma and 3.2 (95%CI 1.42–7.22, p<0.05) for nasal symptoms^33^. Conversely, Duma et al. (2017) found that exposure to QACs did not show a significant association with suboptimal asthma control (OR 1.3, 95%CI 0.97–1.75, p=0.14)^28^. Similary, Duma et al. (2020)^26^ and Mwanga et al.^41^ found that QACs exposure was not associated with health risk. Kobos et al. reported a significant increase in skin disorders and allergic reactions with QAC use, with an OR of 2.49 (95%CI 1.25–4.94, p<0.05) compared to those not using QAC-containing products^35^. Ndlela and Naidoo found an increased risk of respiratory issues among cleaners exposed to QACs, with an OR of 3.44 (95%CI 1.13–10.5, p<0.05) for shortness of breath^43^. Su et al.^48^ found no significant association between QACs and respiratory conditions. Conversely, Patel et al. found that QACs were significantly associated with new asthma onset (OR 1.91, 95%CI 1.10–3.33, p<0.05)^47^.

### 4.7. Quaternary ammonium compounds meta-analysis

The meta-analysis to assess the overall effect of QACs exposure on respiratory conditions included five studies accounting for a total of 9,270 individuals and covering nine adverse effects. Three studies were excluded, with reasons detailed in Table S8. The fixed-effect model yielded an OR of 1.30 (95% CI 1.06–1.60, p=0.01). The random-effects model showed an OR of 1.32 (95% CI 0.86–2.04, p=0.178), with moderate heterogeneity among the included studies (I^2^=46%, τ^2^=0.0999, p=0.06) (Figure 3).

The symmetrical funnel plot and the Egger’s test (p=0.67) suggest no substantial publication bias (Table S9). The meta-regression analysis did not identify any significant moderators impacting the overall effect (Table S10). The leave-one-out analysis indicated that the overall effect estimate remained stable and significant, with the fixed-effect model OR ranging from 1.22 to 1.43, except when Patel et al., and Dumas et al., (2017) were omitted (Table S11).

### 4.8. Other disinfectants

Six studies evaluated the occupational health risks from exposure to other disinfectants. Mwanga et al. found a fourfold increase in ocular-nasal symptoms with frequent alcohol-based product use (OR 4.56). Similar risks were observed for orthophthalaldehyde (OR 3.40), enzymatic cleaners (OR 2.57), and chlorhexidine (OR 1.84)^41^. Su et al. reported asthma risks associated with high-level disinfectants, alcohols, enzymes, formaldehyde, detergents, glass cleaners, and phenolic products^48^. Laborde-Castérot et al. linked EDTA in aerosols to respiratory conditions, with positive nasal provocation tests in 10 of 28 patients, indicating significant occupational hazards^37^. Mac Hovcová et al. found that disinfectants were the most frequent chemical agents causing allergic skin diseases, though specific products were not identified^39^. Similarly, Nettis et al. identified components of disinfectants as major agents inducing occupational allergic contact dermatitis^44^.

### 4.9. Relative odds ratios for disinfectants

We evaluated the RORs of respiratory conditions associated with the use of different disinfectants, using chlorine-based products as the reference. When comparing chlorine-based products to glutaraldehyde, the ROR was 0.84 (95% CI 0.67–1.06, p = 0.002), while when compared to QACs, the ROR was 0.76 (95% CI 0.62–0.94, p = 0.012) (Table S12).

### 4.10. Application methods

Eight studies assessed the occupational health risk related to the use of spray and general disinfection tasks (Table 1).

Lee et al. investigated acute symptoms associated with chemical exposures among cleaning workers. After adjusting for age, sex, and job title, respiratory conditions were significantly associated with cleaning tasks that involved spraying, with an OR of 3.16 (95%CI 1.24–8.04, p<0.05) for medium exposure (duration of exposure per day between 0.5 and 2 hours while wearing PPE most or all of the time). For high exposure (duration of exposure per day exceeding 2 hours without wearing PPE or wearing it rarely) the association was not significant (OR 1.98, 95%CI 0.87–4.51, p>0.05). Additionally, cleaning tasks involving spraying were associated with chemical-related symptoms for workers with high exposure, with an OR of 2.82 (95%CI 1.16–6.82, p<0.05). Other application methods, such as mopping, wet cleaning, and damp wiping, were not significantly associated with chemical-related symptoms or respiratory conditions at medium (OR 2.3, 95%CI 0.74–7.17, p>0.05) and high (OR 3.11, 95%CI 0.94–10.3, p>0.05) exposure. A variety of cleaners, degreasers, finishers, sealers, and polishes were used in the study setting^38^. Caridi et al. investigated the association of asthma and related outcomes with occupations and tasks. The authors found that the task of cleaning and disinfecting fixed surfaces was significantly associated with most outcome variables, including current asthma (OR 1.84, 95%CI 1.26–2.68), moderate exacerbation (OR 3.10, 95% CI 1.25–7.67), and bronchial hyper-responsiveness-related symptoms (OR 1.38, 95% CI 1.08–1.77)^20^. Kurth et al. assessed the prevalence of respiratory conditions and their association with workplace exposures and tasks. The authors concluded that asthma and asthma-like symptoms were significantly associated with cleaning and disinfecting products; and cleaning or disinfecting tasks (prevalence ratio 1.50, 95%CI 1.12–2.02)^36^. Mwanga et al. found that the predominant use of sprays rather than wipes for surface cleaning/disinfection was associated with almost fivefold higher odds (OR 5.01, 95%CI 1.80–13.91, p<0.01) of having a higher asthma symptom score. Similarly, manual sterilization and disinfection of medical instruments was associated with work-related ocular-nasal symptoms (OR 2.92, 95%CI 1.33–6.41, p<0.01). No information on the specific cleaning and disinfectant agents was available^41^. Dumas et al. (2012) investigated the associations between asthma and occupational exposure to cleaning agents in HCWs. Significant associations were observed between current asthma and exposure of moderate to high intensity (at least exposed once a week) to cleaning/disinfecting tasks in general (OR 2.32, 95%CI 1.11–4.86, p<0.001) and use of sprays (OR 2.87, 95%CI 1.02–8.11, p<0.001)^25^. Mehtar et al. found that multiple versus single exposure to chlorine spray was associated with an increase in respiratory (OR 32), eyes (OR 30) and skin conditions (OR 22)^40^. According to Gonzalez et al., new-onset asthma amongst HCWs was significantly associated with general disinfection tasks (OR 4.68, 95%CI 1.08–20.22, p=0.03), dilution of disinfectants (OR 4.56, 95%CI 1.0–20.29, p=0.04). The use of spray was not significantly associated (OR 1.30, 95%CI 0.56–3.04, p-value 0.535) ^33^. Conversely, Patel et al. found that use of spray in surface disinfection was significantly associated with new asthma onset (OR 1.97, 95%CI 1.12–3.47, p<0.05)^47^.

### 4.11. Use of spray meta-analysis

**Figure 4.**
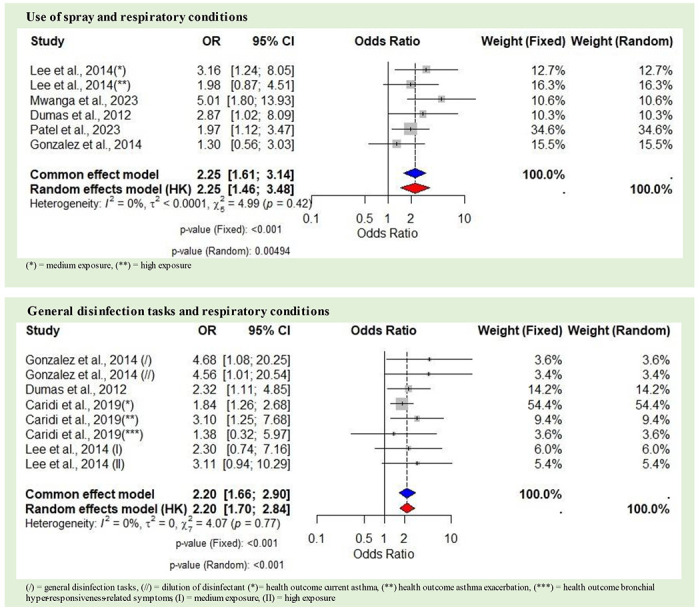
Forest plot showing odds ratios (OR) with 95% confidence intervals (CI) for respiratory conditions associated with application methods. The blue diamond represents the overall common effect estimate, while the red diamond represents the random effect estimate.

The meta-analysis of spray use on respiratory conditions included five studies with 4,568 individuals, addressing six adverse effects. Mehtar et al. was excluded, with reasons detailed in Table S8. The fixed-effect model yielded an OR of 2.25 (95% CI 1.61–3.14, p<0.01). The random-effects model showed an OR of 2.25 (95% CI 1.46–3.48, p=0.004), with non-significant heterogeneity among the included studies (I^2^=0%, τ^2^<0.0001, p=0.42) (Figure 4).

While the funnel plot shows some asymmetry, Egger’s test (p=0.27) indicates that this asymmetry is not statistically significant. (Table S9). The meta-regression analysis did not identify any significant moderators impacting the overall effect (Table S10). The leave-one-out analysis indicated the overall effect estimate remained stable and significant, with the fixed-effect model OR ranging from 2.05 to 2.42 (Table S11).

### 4.12. General disinfection tasks meta-analysis

The meta-analysis of general disinfection tasks on respiratory conditions included four studies, encompassing a total of 3,480 individuals and addressing eight adverse effects. Kurth et al. was excluded, with reasons detailed in Table S8. The fixed-effect model yielded an OR of 2.20 (95% CI 1.66–2.90, p<0.01). The random-effects model showed an OR of 2.20 (95% CI 1.70–2.84, p<0.001), with non-significant heterogeneity among the included studies (I^2^=0%, τ^2^=0, p=0.77) (Figure 4).

The funnel plot exhibited some asymmetry, with a few studies showing larger effect sizes and higher standard errors. Egger’s test yielded a p-value of 0.057. This suggests a borderline significant evidence of publication bias. However, due to the small number of studies, these results should be interpreted with caution (Table S9). The meta-regression analysis did not identify any significant moderators impacting the overall effect (Table S10). The leave-one-out analysis indicated the overall effect estimate remained stable and significant, with the fixed-effect model OR ranging from 2.12 to 2.72 (Table S11).

### 4.13. Relative odds ratios for application methods

We evaluated the ROR of respiratory conditions associated with the general disinfection tasks using use of spray as the reference. The resulting ROR was 0.98 (95% CI 0.74–1.29, p<0.001) (Table S12).

### 4.14. Quality assessment

Among the 30 studies included in the meta-analysis, seven were assessed as having a low risk of bias, while 23 were deemed to have a high risk of bias. The majority of the cross-sectional studies were evaluated as high risk of bias primarily due to the retrospective nature of these studies, where outcomes were determined through self-reported surveys. This method of data collection is common in cross-sectional designs but introduces potential biases related to outcome source and validation.

### 4.15. Mitigation measures

For completeness, we decided to include studies that considered mitigation measures. Specifically, six studies assessed the effect of indoor ventilation on exposure to disinfectant products. Chang et al. evaluated HCWs’ exposure to aerosolized chlorine dioxide during nasoendoscope disinfection in a hospital. Air change rates were adjusted from 4-30 air changes per hour (ACH) to 12-19. Air samples over eight days showed chlorine dioxide concentrations below occupational exposure limits, indicating insignificant exposure in ventilated rooms^22^. Norbäck investigated symptoms among HCWs exposed to glutaraldehyde. Proper ventilation kept glutaraldehyde levels below Swedish occupational exposure limits, while poorly ventilated areas exceeded them. Specific ventilation rates were not available^45^. Lee et al. found that continuous or frequent ventilation reduced the likelihood of respiratory or neurological symptoms in HCWs exposed to chemicals, with an OR of 0.77 (95%CI 0.33-1.76, p<0.05)^38^. Ding et al. examined occupational exposure to high-level disinfectants (HLDs) and miscarriage risk among nurses. Use of gloves and ventilation appeared protective, with a hazard ratio (HR) of 0.9 (95%CI 0.61–1.32)^24^. Nayebzadeh et al. evaluated the impact of work practices and ventilation on peak glutaraldehyde exposure. No correlation was found between ACH and glutaraldehyde levels, suggesting general ventilation alone could not control exposure during solution changeover^42^. Estrin et al. assessed the concentration of ethylene oxide in the breathing zone of HCWs and concluded that it can cause neurological dysfunctions at low concentrations^29^. Multiple studies considered the use of PPE^19,35,44^, but only one quantified the impact. Gaskins et al. assessed the impact of HLDs on fecundity in 1,739 female nurses trying to conceive. HLD-exposed nurses using no PPE, one type of PPE, or two or more types saw conception efforts extended by 18% (95%CI −7–49), 16% (95%CI −3–39), and 0% (95%CI −22–28%), respectively. PPE use ranged from 9% for respiratory protection to 69% for gloves. The study concluded that while HLD exposure correlates with decreased fecundity, PPE use can mitigate this risk. The composition of HLDs was not specified^32^.

## 5 DISCUSSION

This systematic review and meta-analysis assessed the occupational health risks associated with exposure to various disinfectants and application methods among HCWs.

### 5.1. Disinfectants

The meta-analysis examining the occupational health effects of chlorine-based product indicates that exposure to chlorine-based disinfectants is associated with an approximately 71% increase in the odds of developing respiratory conditions. No publication bias was identified, and the leave-one-out analysis confirmed the stability and significance of the overall effect estimate. These results confirm previous findings^49^ regarding the respiratory risks associated with chlorine-based products.

The meta-analysis quantifying the occupational health effect of glutaraldehyde suggests a 44% increase in the odds of respiratory conditions associated with glutaraldehyde exposure. The individual studies included however varied in their findings. Gannon et al. reported occupational asthma at glutaraldehyde levels much lower than current exposure limits^30^, while Gonzalez et al. found no significant association with new-onset asthma^33^. Dumas et al. (2017) linked glutaraldehyde exposure to suboptimal asthma control^28^, but subsequent studies by Dumas et al. (2020, 2021) found no association with asthma incidence^26,27^. These results partially confirm previous findings^50,51^ but highlight the need for further research to clarify the association.

Due to the absence of primary studies with similar exposures and outcomes, a meta-analysis was not performed for products containing peracetic acid, acetic acid, and hydrogen peroxide. However, the individual studies reported variable results. For instance, Casey et al. found that workers with the highest exposure levels to PAA, AA, and HP had a higher prevalence of watery eyes and current asthma^21^. Other studies, such as those by Dalton et al. and Otterspoor and Farrell, found no significant increases in respiratory issues or IgE levels^23,46^. This variability highlights the need for further research.

The meta-analysis quantifying the health effect of QACs suggests a 30% increase in the odds of respiratory conditions associated with QACs exposure. Individual studies presented mixed results. Gonzalez et al. found a significant risk of asthma associated with QACs^33^, whereas Dumas et al. (2017, 2020) found no significant association with suboptimal asthma control or asthma incidence^26,28^. Kobos et al. reported an increased risk of skin disorders and allergic reactions, and Ndlela and Naidoo linked QAC exposure to respiratory issues^43^. These results confirm previous findings^33,43^ on the health risks associated with QACs but also highlight variability in study outcomes highlight the need for further research.

We evaluated the relative odds ratios of respiratory conditions associated with the use of different disinfectants. The ROR for glutaraldehyde was 0.84 (95%CI 0.67-1.06), suggesting 16% lower odds of respiratory conditions compared to chlorine-based products. However, the confidence interval includes 1, indicating that this difference is not statistically significant. The p-value was 0.002, suggesting a significant difference, but the confidence interval’s inclusion of 1 complicates this interpretation.

The ROR for QACs was 0.76 (95%CI 0.62-0.94), indicating 24% lower odds of respiratory conditions compared to chlorine-based products. The confidence interval, which does not include 1, and the p-value of 0.012, indicate a statistically significant difference.

These results indicate that chlorine-based products may pose a higher risk of respiratory conditions compared to glutaraldehyde and QACs. Among the three disinfectants evaluated, QACs were associated with the lowest risk.

### 5.2. Application Methods

The meta-analysis of spraying as an application method demonstrated a strong association with respiratory conditions, with an OR of 2.25. This suggests a 125% increase in the odds of developing respiratory conditions associated with the use of sprays. A similar association was identified for general disinfection tasks, such as wiping, mopping, and disinfectant dilution, among others. These tasks showed an OR of 2.20, indicating a 120% increase in the odds of respiratory conditions associated with general disinfection activities. The ROR was 0.98 (95% CI 0.74–1.29, p<0.001), indicating nearly equal odds of respiratory conditions compared to the use of spray. The confidence interval includes 1, indicating that this difference is not statistically significant. This result indicates the need for further research to explore the specific conditions under which specific application methods might pose greater risks, considering factors like exposure duration, disinfectant concentration, use of PPE, and indoor ventilation. It is important to note that the specific disinfectant products used in studies on spraying and general disinfection tasks were not always identified. Additionally, neither the use of PPE nor details about ventilation were consistently reported in these studies. This lack of information limits the ability to attribute the observed health effects to specific chemicals, the absence of protection, or the application methods alone. Furthermore, considering the retrospective nature of most of the included studies and the pungent odor of many disinfectants, these findings are potentially susceptible to recall bias.

Although respiratory symptoms were the most frequent adverse effects observed, suggesting a critical role played by aerosols and gases released by chemical products during disinfection procedures, few studies applied air sampling to quantitatively evaluate exposure levels. This paucity of quantitative exposure data further complicates the interpretation of the association between disinfectant use and respiratory health outcomes. Although meta-analysis was not possible, all studies consistently concluded that indoor ventilation contribute to reduce the chemical concentration in air and thus mitigating the adverse health effects. A similar protective effect is suggested for PPE.

The cross-sectional design of most studies limited our ability to establish causality between exposure to disinfectants, application methods, and the development of respiratory conditions. Despite this limitation, the findings consistently align with prior research that has documented similar associations. Furthermore, the statistical significance of the results strengthens the evidence that exposure to disinfectants, regardless of the application methods, constitutes an occupational health risk. This consistency across multiple studies underscores the importance of mitigating exposure to disinfectants to protect the respiratory health of workers in various settings.

Our systematic review has several strengths. It evaluated and quantified the respiratory health risk associated with different disinfectants and application methods. Additionally, we assessed the evidence quality by applying a previously validated tool for occupational health studies. The inclusion of both fixed- and random-effects models in the meta-analysis ensured a robust evaluation of the data, accounting for potential variability among studies. Finally, we managed to compare the pooled effect sizes of different disinfectants and application methods. Limitations of this analysis include the exclusion of articles not written in English. Additionally, the potential for misclassification of both exposure and outcomes cannot be ruled out, and not all studies adjusted for the same potential confounders. However, the meta-analyses demonstrated low heterogeneity, allowing for the use of the fixed-effect model. While this study focused on respiratory conditions, other outcomes such as skin and ocular conditions were underreported, limiting the comprehensiveness of the assessment. Variations in exposure assessment methods further complicate comparisons. Moreover, the unspecified disinfectants and the lack of information on PPE and ventilation in studies of addressing the use of spray and general disinfection tasks introduce additional uncertainty.

The findings of this review have significant implications for occupational health policies and practices in healthcare settings. The increased risk posed by chlorine-based products compared to glutaraldehyde and QACs suggests the need to transition toward less hazardous disinfectants. Similarly, the comparable risk associated with the use of sprays and general disinfection tasks highlights the importance of implementing mitigation measures, regardless of the specific application methods. These measures may include the use of appropriate PPE, improved ventilation, and training for workers on safe disinfection practices to minimize exposure and protect respiratory health. Further research, ideally prospective cohorts using precise quantitative exposure assessment, including air sampling, would help clarify both the underlying causal agents and the relevant environmental mechanisms.

## 6 CONCLUSION

Our systematic review found that occupational exposure to chlorine-based products, glutaraldehyde, and QACs is associated with respiratory conditions. We identified chlorine-based products as the most hazardous disinfectants, while QACs were the least hazardous. Similarly, we found that the use of sprays is as dangerous as general disinfection tasks such as wiping, mopping, disinfectant preparation and dilution.

Our findings do not support banning the use of sprays in filovirus outbreak responses, where wiping, being more labor-intensive and time-consuming, may increase the risk of heat stress and other health issues for healthcare workers wearing personal protective equipment. Instead, our results advocate for the recommendation of using less hazardous disinfectant products, such as QACs, coupled with the use of mitigation measures to enhance the safety of disinfection procedures.

## Supplementary Material

Supplement 1

## Figures and Tables

**Table 1.** Data synthesis for included studies

Authors, Year, study design	Risk of bias	Study objective	Type of recruitment, population	Sample size, Sex, age	Exposure (category), assessment	Outcome (cluster) assessment	Adjustment confounding	Main findings
Blackley et al., 2023^19^ Cross-sectional	Low	To assess associations between exposures to PAA, AA, and HP, and work-related eye and airway symptoms	Hospital staff performing cleaning duties and other staff in areas where cleaning occurred	67, female (76%), median age 47 years	Personal or mobile samples for HP, PAA, and AA; additional area samples (PAA, AA, and HP, MM)	Eye, skin, upper and lower airway symptoms assessed via post-shift survey (RC, ON)	Age, gender, smoking status, use other products	PAA, AA, and HP associated with ON and RC
Caridi et al., 2019^20^ Cross-sectional	High	To investigate the association of asthma and related outcomes with occupations and tasks	Members of the Service Employees International Union	2,030, 76% female, average age 48.6 years	Questionnaire on demographic characteristics, tasks performed, products used in healthcare occupations, and occurrence of asthma and related health outcomes (unspecified products)	Post-hire asthma, current asthma, exacerbation of asthma, BHR-related symptoms, asthma score, and wheeze (RC)	Gender, age, race, smoking status, allergies	Surface cleaning associated with RC
Casey et al., 2017^21^ Cross-sectional	High	To assess health effect of PAA, AA, and HP	Current staff of the hospital (volunteers)	163, 50 males, 113 females 49 air samples	Air samples PAA, AA, and HP (PAA, AA, and HP)	Work-related symptoms, questionnaire (RC, ON)	Demographic, smoking status	PAA, AA, and HP associated with ON
Chang et al., 2018^22^ Case report	High	To assess the exposure of HCWs to airborne chlorine dioxide	HCWs who performed nasoendoscope disinfection	14 long-term personal, 4 short-term personal, 16 long-term area samples	ClO_2_ levels measured using ion-chromatograph after collection in midget impingers (Chlorine, MM)	ClO_2_ concentrations were all below the OEL (RC)	N/A	Ventilation mitigates the risk
Dalton et al., 2023^23^ Mix method	Low	To characterize exposures and measures of eye and respiratory tract irritation with PAA, AA, and HP	Volunteers recruited by external company	44, 36 males, 8 females, mean age 40.7 years	Breathing-zone concentrations of PAA, AA, and HP measured using OSHA and NIOSH methods (PAA, AA, and HP)	Tissue injury or inflammation, subjective odor or irritation scores (RC)	N/A	PAA, AA, and HP not associated with ON and RC
Ding et al., 2021^24^ Cohort	High	To examine the association of occupational exposure to HLDs with the risk of miscarriage among nurses	Recruited from the Nurses’ Health Study 3 (NHS3)	2579 nurses with 3974 pregnancies	Self-reported use of HLDs, including GU, orthophthalaldehyde, PAA, AA, and HP; frequency and duration of use; use of exposure controls (MM)	Miscarriage rates obtained from follow-up questionnaires (RC, SC)	Age, education, race, BMI, smoking status, and other exposures	HLDs not associated with miscarriage
Dumas et al., 2012^25^ Cross-sectional	High	To determine the associations between asthma and occupational exposure to cleaning agents	HCWs and a reference population from the French cohort study (EGEA)	543, N/A, 18-79 years	Self-report, expert assessment, and asthma-specific job-exposure matrix (Chlorine, spray, GDTs)	Asthma (RC)	Age, smoking status, BMI	Us of spray associated with asthma
Dumas et al., 2020 ^26^ Cohort	High	To investigate the association between occupational exposure to disinfectants and incident asthma in cohort of U.S. female nurses	Participants from the Nurses’ Health Study II (NHSII)	61,539, mean age 55 years at baseline	Occupational exposure to disinfectants evaluated by questionnaire and JTEM (Chlorine, PAA, AA, HP, GU, QACs)	Incident physician-diagnosed asthma reported during follow-up (RC)	Age, race, ethnicity, smoking status, BMI	Disinfectants not associated with asthma
Dumas et al., 2021^27^ Cohort	High	To investigate the association between use of HLDs and asthma incidence	Participants from the Nurses’ Health Study 3 (NHS3)	17,280, female, mean age 34 years;	Self-reported use of HLDs via questionnaire; duration of use; type of HLDs used in the past month; frequency of PPE use (Chlorine, PAA, AA, HP, GU, QACs)	Incident clinician-diagnosed asthma reported during follow-up (RC)	Age, race, ethnicity, smoking status, BMI	HLDs associated with asthma
Dumas et al., 2017^28^ Cross-sectional	High	To examine the association between occupational exposure to disinfectants and asthma control in U.S. nurses	Participants from the Nurses’ Health Study II (NHSII)	4,102, mean age 58 years; predominantly female	Occupational exposure to disinfectants evaluated by JTEM and self-reported disinfection tasks (Chlorine, PAA, AA, HP, GU, QACs)	Asthma control measured using the Asthma Control Test (RC)	Age, smoking status, BMI, race, ethnicity	Disinfectants associated with poor asthma control
Estrin et al., 1987 ^29^ Case control	High	To detect neurologic effects of chronic low-dose exposure to ethylene oxide	Hospital workers exposed to ethylene oxide and non-exposed controls	8, female, N/A	Hygienic measurements in the breathing zone, personal sampling (MM)	Psychometric test, nerve conduction studies, EEG spectral analysis, standardized neurologic examination (NC)	N/A	Ethylene oxide associated with neurologic dysfunction
Gannon et al., 1995^30^ Case series	High	To investigate cases of occupational asthma due to GU	Workers referred to a specialist occupational lung disease clinic	8, 7 females, 1 male, 29-53 years	Personal and static short and longer-term air samples, specific bronchial provocation tests (GU)	Occupational asthma by PEF measurements and specific bronchial provocation tests (RC)	N/A	GU associated to astham
Garrido et al., 2022^31^ Case control	High	To identify work tasks and cleaning/disinfecting agents associated with respiratory symptoms and hand dermatitis among HCWs in a tertiary hospital	Staff of three hospitals	230 exposed, 80%female, 77 control, 84% female, median age 44 years	Questionnaire on cleaning agent usage, respiratory symptoms, and skin symptoms; frequency of specific tasks and cleaning agents used (Chlorine)	Self-reported respiratory symptoms and hand dermatitis (RC)	Age, sex	Disinfectants associated with RC and skin symptoms
Gaskins et al., 2017^32^ Cohort	High	To examine the relationship between occupational use of HLDs and fecundity among female nurses	Participants from the Nurses’ Health Study 3 (NHS3)	1,739, female, mean age 33.8	Self-reported use of HLDs, frequency and duration of use, and use of PPE (MM)	Duration of pregnancy attempt reported every six months	Age, BMI, smoking status, marital status, race	HLDs associated with reduced fecundity
Gonzalez et al., 2014 ^33^ Cross-sectional	High	To analyze associations between asthma and occupational exposure to disinfectants	Stratified random sampling of various healthcare departments	543, 59 males, 474 females, mean age 39.9 years	Occupational exposure assessment through a work questionnaire, workplace studies (chlorine, GU, QACs, spray)	Asthma, new-onset asthma, nasal symptoms at work, specific IgE assays (RC, ON)	Age, BMI, gender, smoking status, co-exposures	Disinfectant’s dilution and mixing associated with RC
Hawley et al., 2018^34^ Cross-sectional	Low	To assess respiratory symptoms in hospital cleaning staff exposed to PAA, AA, and HP,	Hospital cleaning staff on all three shifts	50, 57% female, median age 40 years	Full-shift samples for HP, PAA, and AA; personal and mobile-area sampling; observation of cleaning tasks (PAA, AA, and HP)	Acute upper and lower airway symptoms from post-shift survey; chronic respiratory symptoms from extended questionnaire	Age, gender, and smoking status	PAA, AA, and HP associated with eye symptoms and RC
Kobos et al., 2022^35^ Cross-sectional	High	To characterize the prevalence of cleaning and disinfection product use, glove use during cleaning and disinfection, and skin/allergy symptoms by occupation	Current employees	559, 77% female, median age 49 years	Questionnaire on cleaning and disinfection product use, glove use, and skin/allergy symptoms (Chlorine, PAA, AA, and HP, QACs, MM)	Prevalence of skin disorders and allergic reactions, glove use frequency (SC)	Age, sex, occupation, and product use frequency	Bleach, alcohol and QACs associated with skin disorders
Kurth et al., 2017^36^ Cross-sectional	High	To estimate the prevalence of current asthma and asthma-like symptoms and their association with workplace exposures and tasks	Convenience sample	562, 78% female, mean age 46.5 years	Questionnaire on respiratory health, work characteristics, tasks performed, products used, and exposures (GDTs)	Self-reported current asthma, asthma-like symptoms, and breathing problems (RC)	Age, sex, race, smoking status, allergy	Disinfection tasks associated with RC
Laborde-Castérot et al., 2012^37^ Case series	High	To report cases of work-related rhinitis and asthma associated with exposure to EDTA-containing detergents or disinfectants	Patients with work-related rhinitis referred for NPT with EDTA	28	History of exposure to aerosols of EDTA-containing products, NPT with tetrasoium EDTA (1-4%)	Positive NPT, presence of rhinitis symptoms, asthma-like symptoms, pulmonary function tests	N/A	EDTA associated with RC
Lee et al., 2014^38^ Cross-sectional	High	To investigate acute symptoms associated with chemical exposures among HCWs work practices	Convenience sample of HCWs employed	183, 81 males, 102 females mean age 48 years	Self-reported data on chemical exposure, tasks performed, and use of PPE (spray, GDTs)	CRS (respiratory, eye, skin, neurological, gastrointestinal), interviews or questionnaires (RC)	age, sex, and job title	Use of spray and disinfectants associated with CRS
Mac Hovcová et al., 2013 ^39^ Cohort	High	To analyze the causes and trends in allergic and irritant-induced skin diseases in the healthcare sector	Data extracted from the National Registry of Occupational Diseases in the Czech Republic from 1997 to 2009	545 95% female, mean age 38 years	Analysis of reported cases of occupational skin diseases, including patch testing and workplace hygiene evaluation	Prevalence and incidence of occupational skin diseases, trends over time, common causative agents	N/A	Disinfectants first cause of allergic skin diseases
Mehtar et al., 2016^40^ Cross-sectional	High	To determine the adverse effects of chlorine spray exposure on humans	Volunteers including HCWs Ebola survivors, and quarantined contacts	1550, 576 males, 974 females, 19-50 years	Self-reported chlorine spray exposure, frequency, and clinical condition post-exposure (chlorine, spray)	Prevalence of eye, respiratory, and skin conditions following chlorine exposure (RC, ON, SC)	Ebola disease effects on eyes	Spray of chlorine associated with eye, skin and RC
Mwanga et al., 2023^41^ Cross-sectional	Low	To investigate occupational risk factors and exposure–response relationships for airway disease among HCWs exposed to cleaning agents	Stratified random sampling	699 77% female, median age 42 years	Self-reported exposure to cleaning agents and related tasks, fractional exhaled nitric oxide testing, blood samples for atopy determination (chlorine, GU, QACs, spray)	ASS, WRONS, WRAS, FeNO levels (ON, RC)	atopy, gender, smoking, age	Disinfectants and use of spray associated with RC
Nayebzadeh, 2007^42^ Mix method	High	To evaluate the impact of work practices and general ventilation systems on HCWs’ peak exposure to GU	HCWs from five hospitals in Quebec, Canada	42 personal samples, 53 HCWs interviewed	Breathing zone personal air samples, classified work practices, presence of local or general ventilation system (GU, MM)	Concentration of GU, exposure levels, prevalence of symptoms like headache and itchy eyes among HCWs	N/A	Work practices affect GU exposure
Ndlela & Naidoo, 2023^43^ Cross-sectional	Low	To investigate the relationship between exposure to cleaning and disinfecting agents and respiratory outcomes	Eligible cleaners from three public hospitals	174, 81% female, mean age 43.2 years	Self-reported frequency and duration of cleaning tasks and agent exposure, skin prick testing, spirometry (Chlorine, QACs)	Respiratory symptoms, chest illnesses (asthma, tuberculosis, hay fever, chronic bronchitis), lung function measures (RC)	Sex, age, smoking history, any allergy, smoke	Disinfectant associated with RC
Nettis et al., 2002^44^ Cohort	High	To determine the prevalence and causes of occupational irritant and allergic contact dermatitis	HCWs referred to the Section of Allergy and Clinical Immunology at the University of Bari from 1994 to 1998	360, 280 females 80 males; mean age 37.8 years	Patch testing with standard series and ‘health’ screening series, additional patch test with rubber allergens when necessary	Positive patch test reactions, diagnoses of allergic and irritant contact dermatitis	N/A	Disinfectants associated with allergic contact dermatitis
Norbäck, 1988^45^ Cross-sectional	Low	To study the prevalence of certain symptoms among HCWs with and without exposure to GU during cold sterilization	HCWs handling GU and a reference group of unexposed workers	107	Hygienic measurements in the breathing zone (GU, MM)	Self-reported symptoms from a questionnaire, including eye, skin, and airway symptoms, headache, nausea, and fatigue (ON, RC)	Demographic data	Ventilation mitigate GU exposure. GU associated with RC
Otterspoor & Farrell, 2019^46^ Case report	High	To evaluate buffered PAA as an alternative to chlorine and HP	NA	20	Assessment of adverse staff reactions, safe-work related incident reporting (PAA, AA, and HP)	Acceptance, cost analysis, efficacy (RC)	N/A	PAA, AA, and HP higher acceptance than chlorine
Patel et al., 2023^47^ Cross-sectional	Low	To examine associations of cleaning tasks and products with WRAS in HCWs in Texas in 2016, comparing them to prior results from 2003	Representative sample of Texas HCWs from state licensing boards	2,421, 83% female, average age 48.8 years;	Self-reported data on cleaning tasks, products used, and occupational exposures (Chlorine, GU, QACs, spray)	Self-reported physician-diagnosed asthma, new onset asthma, work-exacerbated asthma, and bronchial hyperresponsiveness	Age, gender, race, atopy, obesity, smoking status, and years on the job	Use of spray, bleach, QACs associated with WRAS
Su et al., 2019^48^ Cross-sectional	High	To identify and group HCWs with similar patterns of asthma symptoms and explore their associations with patterns of cleaning and disinfecting activities (CDAs)	HCWs from nine selected occupations	2029, 1542 females, 487 males, N/A	Self-reported information on asthma symptoms/care, CDAs, demographics, smoking status, allergic status (chlorine, QACs)	Asthma symptoms clusters and their associations with exposure clusters (ECs) through multinomial logistic regression (RC)	Age, gender, education, smoking status, and allergic status	Chlorine associated with RC

N/A = Not available, NA = Not applicable, HCWs = Healthcare workers, OEL = Occupational exposure limits, PAA = Peracetic acid, AA = Acetic acid, HP = Hydrogen peroxide, GU = glutaraldehyde, PEF = peak expiratory flow, RC = respiratory conditions, MM = Mitigation measure, ON = Ocular-nasal conditions, SC = Skin conditions, GDTs = General disinfection tasks, QACs = Quaternary ammonium compounds, PPE = personal protective equipment, CRS = Chemical-related symptoms, ASS = Asthma Symptom Score, WRONS = work-related ocular-nasal symptoms, WRAS = work-related asthma symptoms, HLDs = high-level disinfectants, BMI = body mass index, JTEM = job-task-exposure matrix, NPT = nasal provocation test, NC = Neurological conditions

## Data Availability

All data relevant to the study are included in the article or available as supplementary materials.

## Article summary

### Strengths and limitations of this study

- The systematic review and meta-analysis provide a comprehensive comparison of the health effects of occupational exposure to various disinfectants and application methods in healthcare settings.
- The study includes a broad range of disinfectants and application methods, offering a detailed assessment that extends beyond filovirus treatment centers.
- The inclusion of both fixed- and random-effects models in the meta-analysis ensures a robust evaluation of the data, accounting for potential variability among studies.
- While this study focused on respiratory conditions, other outcomes such as skin and ocular conditions were underreported, limiting the comprehensiveness of the assessment.
- Variations in exposure assessment methods and the lack of information on specific disinfectant products, PPE use, and ventilation further complicate comparisons and limit the ability to attribute health effects to specific factors.

## References

[1] International Committee on Taxonomy of Viruses. Genus: Orthoebolavirus | ICTV. The ICTV Report on Virus Classification and Taxon Nomenclature. Published 2024. Accessed April 12, 2024. https://ictv.global/report/chapter/filoviridae/filoviridae/orthoebolavirus

[2] World Health Organization, Centers for Disease Control and Prevention. Infection Control for Viral Haemorrhagic Fevers in the African Health Care Setting.; 1998. Accessed March 2, 2019. https://www.cdc.gov/vhf/abroad/pdf/african-healthcare-setting-vhf.pdf

[3] World Health Organization. Ebola Guidance Package Infection Prevention and Control (IPC) Guidance Summary Background.; 2014. Accessed March 4, 2019. http://www.who.int/csr/resources/publications/ebola/filovirus_infection_control/en/

[4] Carpenter A, Cox AT, Marion D, Phillips A, Ewington I. A case of a chlorine inhalation injury in an Ebola treatment unit. J R Army Med Corps. 2016;162(3):229–231. doi:10.1136/jramc-2015-000501 26472120

[5] Mehtar S, Bulabula ANH, Nyandemoh H, Jambawai S. Deliberate exposure of humans to chlorine-the aftermath of Ebola in West Africa. Antimicrob Resist Infect Control. 2016;5(1):1–8. doi:10.1186/s13756-016-0144-1 27895903 PMC5109677

[6] World Health Organization. Infection Prevention and Control Guideline for Ebola and Marburg Disease.; 2023. https://www.who.int/publications/i/item/WHO-WPE-CRS-HCR-2023.1 42313992

[7] Quinn TD, Kim JH, Strauch A, Physiological Evaluation of Cooling Devices in Conjunction With Personal Protective Ensembles Recommended for Use in West Africa. Disaster Med Public Health Prep. 2017;11(5):573–579. doi:10.1017/dmp.2016.209 28303772 PMC9903158

[8] Zhu Y, Mao Y, Li Y, Field Investigation of the Heat Stress in Outdoor of Healthcare Workers Wearing Personal Protective Equipment in South China. Front Public Heal. 2023;11. doi:10.3389/fpubh.2023.1166056 PMC10151780 37143989

[9] Shamseer L, Moher D, Clarke M, Preferred reporting items for systematic review and meta-analysis protocols (PRISMA-P) 2015: elaboration and explanation. BMJ. 2015;349. doi:10.1136/BMJ.G7647 25555855

[10] Morgan RL, Whaley P, Thayer KA, Schunemann HJ. Identifying the PECO: A framework for formulating good questions to explore the association of environmental and other exposures with health outcomes. Environ Int. 2018;121:1027–1031. doi:10.1016/j.envint.2018.07.015 30166065 PMC6908441

[11] Shamliyan TA, Kane RL, Ansari MT, Development quality criteria to evaluate nontherapeutic studies of incidence, prevalence, or risk factors of chronic diseases: pilot study of new checklists. J Clin Epidemiol. 2011;64(6):637–657. doi:10.1016/J.JCLINEPI.2010.08.006 21071174

[12] Ijaz S, Verbeek J, Seidler A, Night-shift work and breast cancer – a systematic review and meta-analysis. Scand J Work Environ Health. 2013;39(5):431–447. doi:10.5271/sjweh.3371 23804277

[13] Starke KR, Kofahl M, Freiberg A, Are Daycare Workers at a Higher Risk of Parvovirus B19 Infection? A Systematic Review and Meta-Analysis. Int J Environ Res Public Heal 2019, Vol 16, Page 1392. 2019;16(8):1392. doi:10.3390/IJERPH16081392 PMC6517978 30999694

[14] Scottish Intercollegiate Guidelines Network. Checklists. Methodology checklist. Published 2021. Accessed July 5, 2024. https://www.sign.ac.uk/using-our-guidelines/methodology/checklists/

[15] Critical Appraisal Skills Program. CASP Checklist: Systematic Reviews of Observational Studies. CASP Checklist: Systematic Reviews of Observational Studies. Published 2006. Accessed July 5, 2024. https://casp-uk.net/casp-tools-checklists/systematic-reviews-meta-analysis-observational-studies/

[16] Cochrane Training. Identifying and measuring heterogeneity. Cochrane Handbook for Systematic Reviews of Interventions. Published 2024. Accessed July 10, 2024. https://training.cochrane.org/handbook/current/chapter-10#section-10-10-2

[17] Viechtbauer W. Conducting Meta-Analyses in R with the metafor Package. J Stat Softw. 2010;36(3 SE-Articles):1–48. doi:10.18637/jss.v036.i03

[18] Moher D, Liberati A, Tetzlaff J, Preferred reporting items for systematic reviews and meta-analyses: The PRISMA statement. PLoS Med. 2009;6(7). doi:10.1371/journal.pmed.1000097 PMC2707599 19621072

[19] Blackley BH, Nett RJ, Cox-Ganser JM, Harvey RR, Virji MA. Eye and airway symptoms in hospital staff exposed to a product containing hydrogen peroxide, peracetic acid, and acetic acid. Am J Ind Med. 2023;66(8):655–669. doi:10.1002/AJIM.23488 37221450 PMC10431326

[20] Caridi MN, Humann MJ, Liang X, Occupation and task as risk factors for asthma-related outcomes among healthcare workers in New York City. Int J Hyg Environ Health. 2019;222(2):211– 220. doi: 10.1016/J.IJHEH.2018.10.001 30327176 PMC6856954

[21] Casey ML, Hawley B, Edwards N, Cox-Ganser JM, Cummings KJ. Health problems and disinfectant product exposure among staff at a large multispecialty hospital. Am J Infect Control. 2017;45(10):1133–1138. doi:10.1016/J.AJIC.2017.04.003 28549881 PMC5685540

[22] Chang YB, Lee FY, Goh MM, Lam DKH, Tan ABH. Assessment of occupational exposure to airborne chlorine dioxide of healthcare workers using impregnated wipes during high-level disinfection of non-lumened flexible nasoendoscopes. J Occup Environ Hyg. 2018;15(12):818–823. doi:10.1080/15459624.2018.1523617 30215576

[23] Dalton PH, Maute C, Hicks JB, Watson HN, Loccisano AE, Kerger BD. Environmental chamber studies of eye and respiratory irritation from use of a peracetic acid-based hospital surface disinfectant. Antimicrob Steward Healthc Epidemiol. 2023;3(1). doi:10.1017/ASH.2023.138 PMC10127244 37113200

[24] Ding M, Lawson C, Johnson C, Occupational exposure to high-level disinfectants and risk of miscarriage among nurses. Occup Environ Med. 2021;78(10):731–737. doi:10.1136/QEMED-2020-107297 34039757 PMC8634624

[25] Dumas O, Donnay C, Heederik DJJ, Occupational exposure to cleaning products and asthma in hospital workers. Occup Environ Med. 2012;69(12):883–889. doi:10.1136/OEMED-2012-100826 23033509

[26] Dumas O, Boggs KM, Quinot C, Occupational exposure to disinfectants and asthma incidence in U.S. nurses: A prospective cohort study. Am J Ind Med. 2020;63(1):44–50. doi:10.1002/AJIM.23067 31692020 PMC6891131

[27] Dumas O, Gaskins AJ, Boggs KM, Occupational use of high-level disinfectants and asthma incidence in early to mid-career female nurses: a prospective cohort study. Occup Environ Med. 2021;78(4):244. doi:10.1136/OEMED-2020-106793 33452037 PMC7985390

[28] Dumas O, Wiley AS, Quinot C, Occupational exposure to disinfectants and asthma control in US nurses. Eur Respir J. 2017;50(4). doi:10.1183/13993003.00237-2017 PMC5702691 28982772

[29] Estrin WJ, Cavalieri SA, Wald P, Becker CE, Jones JR, Cone JE. Evidence of Neurologic Dysfunction Related to Long-term Ethylene Oxide Exposure. Arch Neurol. 1987;44(12):1283–1286. doi:10.1001/ARCHNEUR.1987.00520240057012 3314817

[30] Gannon PFG, Bright P, Campbell M, O’Hickey SP, Sherwood Burge P. Occupational asthma due to glutaraldehyde and formaldehyde in endoscopy and x ray departments. Thorax. 1995;50(2):156159. doi:10.1136/THX.50.2.156 PMC473910 7701454

[31] Garrido AN, House R, Lipszyc JC, Liss GM, Holness DL, Tarlo SM. Cleaning agent usage in healthcare professionals and relationship to lung and skin symptoms. J Asthma. 2022;59(4):673–681. doi:10.1080/02770903.2021.1871740 33402006

[32] Gaskins AJ, Chavarro JE, Rich-Edwards JW, Occupational use of high-level disinfectants and fecundity among nurses. Scand J Work Environ Heal. 2017;43(2):171–180. doi:10.5271/SJWEH.3623 PMC5840865 28125764

[33] Gonzalez M, Jegu J, Kopferschmitt MC, Asthma among workers in healthcare settings: Role of disinfection with quaternary ammonium compounds. Clin Exp Allergy. 2014;44(3):393–406. doi:10.1111/cea.12215 24128009

[34] Hawley B, Casey M, Virji MA, Cummings KJ, Johnson A, Cox-Ganser J. Respiratory symptoms in hospital cleaning staff exposed to a product containing hydrogen peroxide, peracetic acid, and acetic acid. Ann Work Expo Heal. 2018;62(1):28–40. doi:10.1093/ANNWEH/WXX087 PMC5757516 29077798

[35] Kobos L, Anderson K, Kurth L, Characterization of Cleaning and Disinfection Product Use, Glove Use, and Skin Disorders by Healthcare Occupations in a Midwestern Healthcare Facility. Buildings. 2022;12(12). doi:10.3390/BUILDINGS12122216 PMC11034745 38650891

[36] Kurth L, Virji MA, Storey E, Current asthma and asthma-like symptoms among workers at a Veterans Administration Medical Center. Int J Hyg Environ Health. 2017;220(8):1325–1332. doi:10.1016/j.ijheh.2017.09.001 28923472 PMC5965269

[37] Laborde-Castérot H, Villa AF, Rosenberg N, Dupont P, Lee HM, Garnier R. Occupational rhinitis and asthma due to EDTA-containing detergents or disinfectants. Am J Ind Med. 2012;55(8):677–682. doi:10.1002/AJIM.22036 22431256

[38] Lee SJ, Nam B, Harrison R, Hong O. Acute symptoms associated with chemical exposures and safe work practices among hospital and campus cleaning workers: A pilot study. Am J Ind Med. 2014;57(11):1216–1226. doi:10.1002/AJIM.22376 25223949 PMC9275785

[39] Mac Hovcová A, Fenclová Z, Pelclová D. Occupational skin diseases in Czech healthcare workers from 1997 to 2009. Int Arch Occup Environ Health. 2013;86(3):289–294. doi:10.1007/S00420-012-0764-6/FIGURES/2 22466250

[40] Mehtar S, Bulabula ANH, Nyandemoh H, Jambawai S. Deliberate exposure of humans to chlorine- the aftermath of Ebola in West Africa. Antimicrob Resist Infect Control. 2016;5(1). doi:10.1186/S13756-016-0144-1 PMC5109677 27895903

[41] Mwanga HH, Baatjies R, Jeebhay MF. Occupational risk factors and exposure-response relationships for airway disease among health workers exposed to cleaning agents in tertiary hospitals. Occup Environ Med. Published online 2023. doi:10.1136/OEMED-2022-108763 37137692

[42] Nayebzadeh A. The effect of work practices on personal exposure to glutaraldehyde among health care workers. Ind Health. 2007;45(2):289–295. doi:10.2486/INDHEALTH.45.289 17485873

[43] Ndlela NH, Naidoo RN. Job and exposure intensity among hospital cleaning staff adversely affects respiratory health. Am J Ind Med. 2023;66(3):252–264. doi:10.1002/AJIM.23456 36611285

[44] Nettis E, Colanardi MC, Soccio AL, Ferrannini A, Tursi A. Occupational irritant and allergic contact dermatitis among healthcare workers. Contact Dermatitis. 2002;46(2):101–107. doi:10.1034/J.1600-0536.2002.460208.X 11918604

[45] Norback D. Skin and respiratory symptoms from exposure to alkaline glutaraldehyde in medical services. Scand J Work Environ Health. 1988;14(6):366–71. Accessed November 17, 2023. https://www.jstor.org/stable/40965592 2975045

[46] Otterspoor S, Farrell J. An evaluation of buffered peracetic acid as an alternative to chlorine and hydrogen peroxide based disinfectants. Infect Dis Heal. 2019;24(4):240–243. doi:10.1016/J.IDH.2019.06.003 31288991

[47] Patel J, Gimeno Ruiz de Porras D, Mitchell LE, Cleaning Tasks and Products and Asthma Among Healthcare Professionals. J Occup Environ Med. Published online October 6, 2023. doi:10.1097/JOM.0000000000002990 PMC10841035 37801602

[48] Su FC, Friesen MC, Humann M, Clustering asthma symptoms and cleaning and disinfecting activities and evaluating their associations among healthcare workers. Int J Hyg Environ Health. 2019;222(5):873–883. doi:10.1016/J.IJHEH.2019.04.001 31010790 PMC6883647

[49] Starke KR, Friedrich S, Schubert M, Are Healthcare Workers at an Increased Risk for Obstructive Respiratory Diseases Due to Cleaning and Disinfection Agents ? A Systematic Review and Meta-Analysis. Published online 2021. https://www.ncbi.nlm.nih.gov/pmc/articles/PMC8152277/#:~:text=The meta-analysisestimated an,)and instruments (ES % 3D 1.34 10.3390/ijerph18105159 PMC8152277 34068014

[50] Pearlman O. Reviewing the Use of Glutaraldehyde for High-level Disinfection by Sonographers. J Diagnostic Med Sonogr. 2018;35(1):49–57. doi:10.1177/8756479318813361

[51] Wang Y, Wu Q, Ren B, Subacute Pulmonary Toxicity of Glutaraldehyde Aerosols in a Human In Vitro Airway Tissue Model. Int J Mol Sci 2022, Vol 23, Page 12118. 2022;23(20):12118. doi:10.3390/IJMS232012118 PMC9603730 36292975

