# Filovirus outbreak responses and occupational health effects of chlorine spraying in healthcare workers: a systematic review and meta-analysis of alternative disinfectants and application methods

**DOI:** 10.1101/2024.09.18.24313940

**Published:** 2025-02-24

**Authors:** Luca Fontana, Luca Stabile, Elisa Caracci, Antoine Chaillon, Kamal Ait-Ikhlef, Giorgio Buonanno

**Affiliations:** a: Università degli studi di Cassino e del Lazio Meridionale (UNICAS), Department of Civil and Mechanical Engineering, Cassino, Italy.; b: World Health Organization, Geneva, Switzerland.; c: University of California, Center for AIDS Research (CFAR), San Diego. USA.

The authors have withdrawn this manuscript because of data issues that affect the validity of the findings. Therefore, the authors do not wish this work to be cited as reference for the project. If you have any questions, please contact the corresponding author.

